# Reply to Skalidis et al.

**DOI:** 10.1152/ajpheart.00425.2025

**Published:** 2025-10-01

**Authors:** Jussara M. do Carmo, Ana C. M. Omoto, John E. Hall, Xuemei Dai, Emily C. Ladnier, Marilia C. Mouro, Odecio E. S. Tosta, Zhen Wang, Xuan Li, Alexandre A. da Silva

**Affiliations:** 1 Department of Physiology and Biophysics, Mississippi Center for Obesity Research, Cardiorenal and Metabolic Diseases Research Center, University of Mississippi Medical Center, Jackson, Mississippi, United States; 2 Centro Universitário Barão de Mauá, Ribeirão Preto, Brazil

REPLY: We are grateful to Dr. Skalidis et al. (1) for their thoughtful and insightful comments regarding our recent paper on the impact of parental obesity on post-myocardial infarction (MI) outcomes in nonobese offspring (2). They highlight three important areas where there are still gaps in our understanding that deserve further investigation. We agree with their comments and are currently addressing some of these gaps with additional studies.

## CONNEXIN 43 AND SEX-SPECIFIC POST-MI REMODELING

We agree that the selective reduction in connexin 43 (Cx43) expression in male offspring at baseline is an intriguing observation. Although our study did not directly assess arrhythmias, the potential link between reduced Cx43 and increased susceptibility to post-MI arrhythmogenesis, particularly in a sex-specific manner, is plausible. Future studies using telemetry and programmed electrical stimulation may help clarify whether altered gap junction protein expression contributes to the increased mortality we observed in nonobese offspring of obese parents (HFD-Offs), particularly in males.

## MITOCHONDRIAL DYSFUNCTION AND CARDIAC METABOLIC FLEXIBILITY

Dr. Skalidis et al.’s (1) point regarding mitochondrial metabolic flexibility is well-taken. Our findings of impaired carbohydrate-supported oxidative phosphorylation and increased reactive oxygen species (ROS) production in both sexes suggest a preexisting metabolic vulnerability. Investigating whether targeted interventions, such as exercise training (3), pharmacological agents (4), or dietary modifications (5), may improve mitochondrial efficiency and thereby attenuate susceptibility to MI-induced injury is a promising avenue that we are actively exploring in ongoing studies.

## PREEXISTING DIASTOLIC DYSFUNCTION AND LONG-TERM REMODELING

We appreciate the interest in our finding of early diastolic dysfunction in HFD-Offs (6) and agree that chronic follow-up models could provide valuable insights into the progression of cardiac remodeling. Although pulmonary congestion markers were not elevated at the time points evaluated, more severe maladaptive cardiac remodeling may occur over longer periods of time. Future longitudinal studies incorporating pressure-volume loop analyses and serial imaging may help to address this question.

We thank the authors for their kind remarks. Their questions highlight broader implications of our findings and underscore the need for continued exploration of the mechanisms underlying developmental programming of cardiovascular disease.
